# Effects of Commonly Used Pesticides in China on the Mitochondria and Ubiquitin-Proteasome System in Parkinson’s Disease

**DOI:** 10.3390/ijms18122507

**Published:** 2017-11-23

**Authors:** Tingting Chen, Jieqiong Tan, Zhengqing Wan, Yongyi Zou, Henok Kessete Afewerky, Zhuohua Zhang, Tongmei Zhang

**Affiliations:** 1 State Key Laboratory of Medical Genetics, Xiangya Medical School, Central South University, Changsha 410078, China; chentingting@sklmg.edu.cn (T.C.); jieqiong.tan@gmail.com (J.T.); wanzhengqing@sklmg.edu.cn (Z.W.); zouyongyi@gmail.com (Y.Z.); henokessete@hust.edu.cn (H.K.A.); zhangzhuohua@sklmg.edu.cn (Z.Z.); 2 Department of Physiology, School of Basic Medicine, Tongji Medical College, Huazhong University of Science and Technology, Wuhan 430030, China; 3 The Institute of Brain Research, Huazhong University of Science and Technology, Wuhan 430030, China

**Keywords:** Parkinson’s disease, pesticides, mitochondria, ubiquitin-Proteasome system, China

## Abstract

Evidence continues to accumulate that pesticides are the leading candidates of environmental toxins that may contribute to the pathogenesis of Parkinson’s disease. The mechanisms, however, remain largely unclear. According to epidemiological studies, we selected nine representative pesticides (paraquat, rotenone, chlorpyrifos, pendimethalin, endosulfan, fenpyroximate, tebufenpyrad, trichlorphon and carbaryl) which are commonly used in China and detected the effects of the pesticides on mitochondria and ubiquitin-proteasome system (UPS) function. Our results reveal that all the nine studied pesticides induce morphological changes of mitochondria at low concentrations. Paraquat, rotenone, chlorpyrifos, pendimethalin, endosulfan, fenpyroximate and tebufenpyrad induced mitochondria fragmentation. Furthermore, some of them (paraquat, rotenone, chlorpyrifos, fenpyroximate and tebufenpyrad) caused a significant dose-dependent decrease of intracellular ATP. Interestingly, these pesticides which induce mitochondria dysfunction also inhibit 26S and 20S proteasome activity. However, two out of the nine pesticides, namely trichlorphon and carbaryl, were found not to cause mitochondrial fragmentation or functional damage, nor inhibit the activity of the proteasome, which provides significant guidance for selection of pesticides in China. Moreover, our results demonstrate a potential link between inhibition of mitochondria and the UPS, and pesticide-induced Parkinsonism.

## 1. Introduction

Parkinson’s disease (PD) is a progressive neurodegenerative movement disorder affecting approximately 2% of people aged over 65. It is pathologically characterized by pronounced loss of dopaminergic neurons in the substantia nigra of the midbrain and formation of ubiquitin-positive Lewy body aggregates [1,2]. Epidemiological studies indicate both environmental neurotoxins and genetic predisposition as potential risk factors for PD. Evidence continues to accumulate that pesticides are the leading candidates of environmental toxins that may contribute to the pathogenesis of PD [3,4]. Several pesticides such as rotenone, paraquat, dieldrin and maneb have been used to develop PD models, with pathological features of the degeneration of dopaminergic neurons [5,6,7,8,9].

Although the mechanisms of loss of dopaminergic neurons in PD remain unclear, there is compelling evidence that mitochondria and ubiquitin-Proteasome system (UPS) dysfunction represent critical events [10,11]. Identification of mutants in *PINK1*, *DJ-1*, *PRKN* and *LRRK-2* genes, which participate in oxidative stress and mitochondrial dysfunction, affirms the hypotheses of dopaminergic neuronal degeneration in PD [12]. Mutants of α-synuclein, Ubiquitin C-Terminal Hydrolase L1 (UCH-L1) and parkin support the involvement of UPS dysfunction in PD [12,13,14,15,16]. Furthermore, various Parkinsonian toxicants have been shown to impair mitochondria and UPS function. Rotenone, an inhibitor of mitochondrial complex I, demonstrates many features of PD including selective dopaminergic degeneration, increased oxidative damage, Lewy body-like inclusions formation and α-synuclein aggregation [9,17,18]. In addition, exposure to proteasome inhibitors in rats causes motor dysfunction, loss of dopaminergic neurons and formation of Lewy bodies [19]. All these findings are suggestive of the critical role of mitochondria and UPS dysfunction in PD.

Previous studies have linked individual pesticides to mitochondrial dysfunction [20,21,22,23,24] or UPS dysfunction [25,26,27,28,29]. For example, paraquat, rotenone, pyridaben, fenpyroximate, fenazaquin and tebufenpyrad have been reported to directly inhibit complex I [24,30]. Rotenone, ziram, diethyldithiocarbamate, endosulfan, benomyl, cyanazine, dieldrin, metam, propargite, triflumizole and dieldrin showed inhibitory effects on proteasome activities [26,27,29]. However, the correlation between mitochondrial dysfunction and UPS dysfunction for pesticides treatment is not very clear.

In the present study, we have characterized the toxic potency of nine (paraquat, rotenone, chlorpyrifos, pendimethalin, endosulfan, fenpyroximate, tebufenpyrad, trichlorphon and carbaryl) commonly used pesticides that belong to different chemical groups and defined their toxicity mechanism. Our results showed that paraquat, rotenone, chlorpyrifos, pendimethalin, endosulfan, fenpyroximate and tebufenpyrad induce mitochondria fragmentation. Furthermore, some of them (paraquat, rotenone, chlorpyrifos, fenpyroximate and tebufenpyrad) cause a significant dose-dependent decrease of intracellular ATP. However, two out of the nine pesticides, namely trichlorphon and carbaryl, were found not to induce mitochondrial fragmentation or functional damage, nor inhibit the activity of the proteasome. Interestingly, our results suggest that the pesticides that induce mitochondria dysfunction also inhibit 26S and 20S proteasome activity. We also herewith establish a potential link between mitochondria and the UPS.

## 2. Results

### 2.1. Pesticides Caused Dose-Dependent Apoptotic Cell Death in SH-SY5Y Cells

Environmental factors are closely related to the occurrence of PD, and hence researchers have been conducting extensive screening of environmentally predisposing factors in PD, and have found that exposure to pesticides, drinking well water and farming may increase the risk of PD [31]. Among the environmental factors, pesticides, which are widely used throughout the world, are the common factors. Pesticides can be grouped in different ways; according to purposes, pesticides can be divided into insecticides, rodenticides, fungicides, herbicides and so on, and in terms of chemical structures, they can be divided into organophosphate, carbamate, organochlorine, pyrethroid and pseudo chrysanthemum bug [32]. In reference to former epidemiological studies, for the current study, we selected nine representative pesticides (paraquat, rotenone, chlorpyrifos, pendimethalin, endosulfan, fenpyroximate, tebufenpyrad, trichlorphon and carbaryl), which are commonly used in China, and have analyzed their possible toxicological mechanisms to cause cell death.

**Figure 1:**
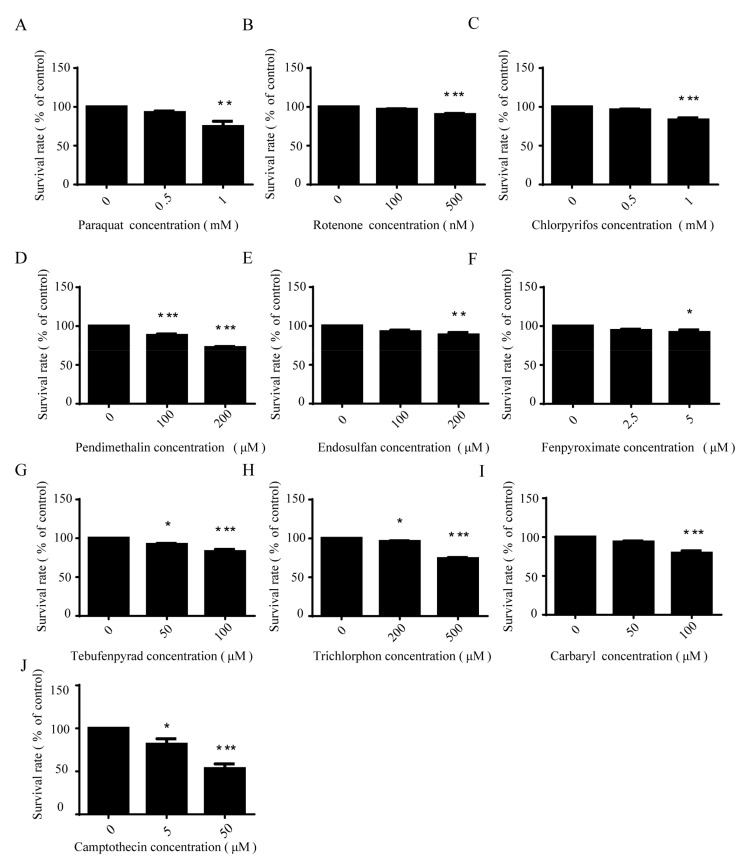
Pesticides caused dose-dependent apoptotic cell death in SH-SY5Y cells. SH-SY5Y cells were treated with different concentrations of pesticides for 24 h. Cell apoptosis (**A**–**I**) was detected using annexin V-FITC/PI assay and analyzed by one-way ANOVA and Dunnett’s test (*n* = 4), and camptothecin (**J**) was the positive control. Error bars represent SEM. * *p* < 0.05; ** *p* < 0.01; *** *p* < 0.001.

The toxicities of pesticides are classified by their lethal concentrations according to WHO classification [33], so we treated SH-SY5Y cells with the proper concentration according to the toxicity of the different pesticides. After being treated with pesticides in different concentrations for 24 h, SH-SY5Y cells were stained with annexin V-FITC and propidium iodide, followed by FACScan flow cytometer analysis. Results showed that these nine pesticides can lead to dose-dependent apoptosis, whereby the lethal concentrations of these pesticides are different, presumably due to the differences in the mechanisms of their toxicity. In general, a low concentration of these pesticides was found to have a toxic, but not lethal, effect on cells. Therefore, in order to study the toxicological mechanism of these pesticides, we chose lower concentrations to carry out the following experiments (Figure 1).

### 2.2. Pesticides Induced Morphological Changes of Mitochondria

**Figure 2:**
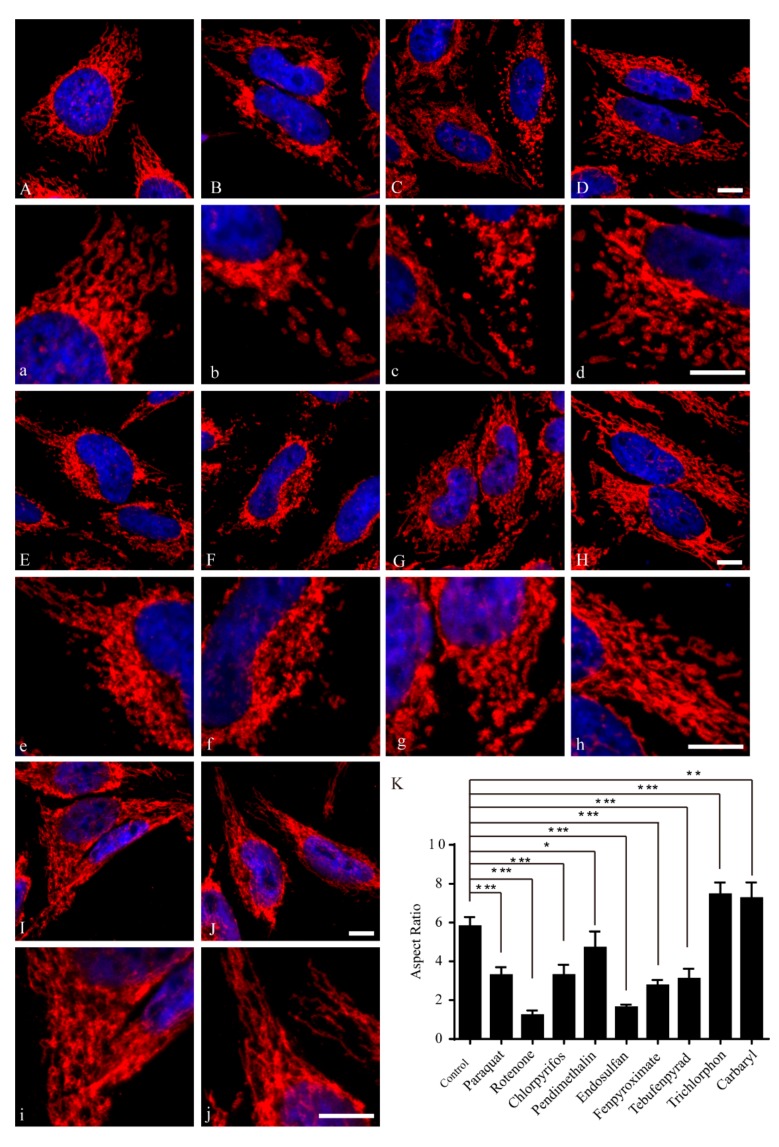
Pesticides induced morphological changes of mitochondria. HeLa cells were treated with different concentrations of pesticides or control solvent DMSO (**A**,**a**) for 24 h. Mitochondria (red) and cell nuclei (blue) are shown. High-magnification pictures (**a**–**j**) of cells are also shown to exemplify the mitochondria. Mitochondria were fragmented after exposure to 500 μM paraquat (**B**,**b**); 1 nM rotenone (**C**,**c**); 200 μM chlorpyrifos (**D**,**d**); 50 μM pendimethalin (**E**,**e**); 50 μM endosulfan (**F**,**f**); 1 μM fenpyroximate (**G**,**g**) and 20 μM tebufenpyrad (**H**,**h**). Conversely, mitochondria were elongated after exposure to 200 μM trichlorphon (**I**,**i**) and 100 μM carbaryl (**J**,**j**); bar = 10 μM. Mitochondrial aspect ratios in control or different pesticide-treated cells were measured by ImageJ and analyzed by one-way ANOVA and Dunnett’s test (**K**). The aspect ratios of mitochondria were analyzed by one-way ANOVA and Dunnett’s test (*n* = 50 cells); error bars represent SEM. * *p* < 0.05; ** *p* < 0.01; *** *p* < 0.001.

Several pesticides are known to cause neurotoxicological effects in humans. For example, exposure to the most common pesticides, namely rotenone and paraquat, induces behavioral and pathological changes that characterize PD in animal models and humans [24,34]. Moreover, the potential mechanism of these pesticides may increase the risk of PD through disruption of mitochondrial function [35]. Recent studies revealed that the mitochondrial morphology is an important determinant of mitochondrial function [36,37]. To investigate the effect of pesticide on mitochondrial morphology, HeLa cells were treated with different concentrations of various pesticides for 24 h. Cells were then fixed and labeled with a specific antibody against mitochondrial outer membrane protein TOM20, and immunolocalization was visualized by laser confocal microscopy. Mitochondria demonstrated a tubular and filamentous morphology in control HeLa cells. We measured mitochondrial aspect ratio to get the changes of the length of mitochondria. Aspect ratio (an index of mitochondrial branch length) means the ratio between the bigger (major) and the smaller (minor) side of each mitochondrial fragment. The results showed that seven out of nine representative pesticides, including paraquat (500 μM), rotenone (1 nM), chlorpyrifos (200 μM), pendimethalin (50 μM), endosulfan (50 μM), fenpyroximate (1 μM) and tebufenpyrad (20 μM), induced mitochondrial fragmentation to varying degrees (Figure 2A–H,K). However, trichlorphon (200 μM) and carbaryl (100 μM) caused mitochondrial elongation (Figure 2I–K). As for nuclear shape, no difference was noted between pesticide-treated and control cells. These results suggest that pesticides induce morphological changes of mitochondria prior to cell death.

### 2.3. Paraquat, Rotenone, Chlorpyrifos, Fenpyroximate and Tebufenpyrad Induced a Significant Dose-Dependent Decrease of Intracellular ATP

**Figure 3:**
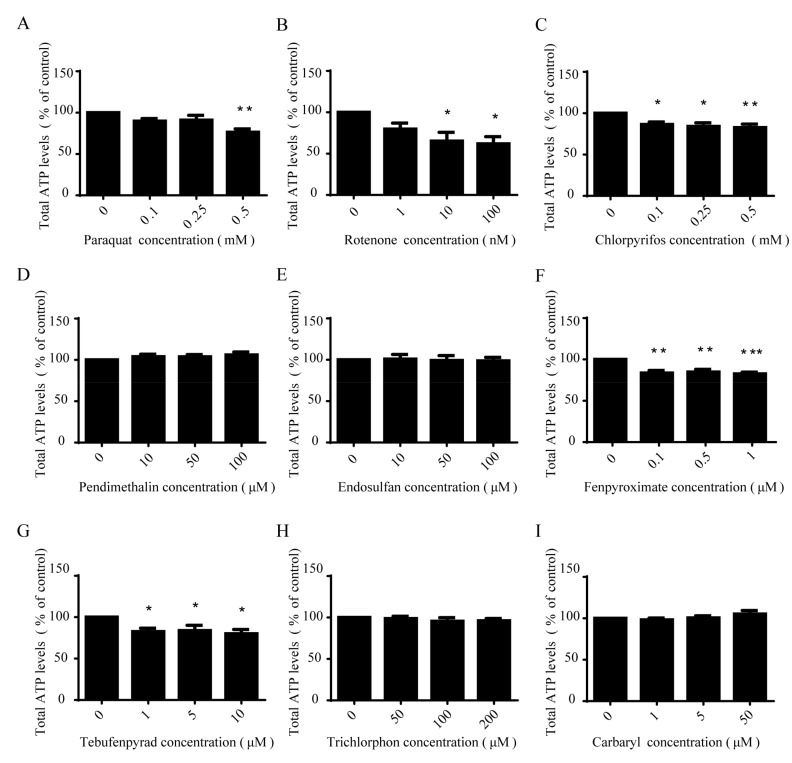
Paraquat, rotenone, chlorpyrifos, fenpyroximate and tebufenpyrad induced a significant dose-dependent decrease of intracellular ATP. SH-SY5Y cells were exposed to low concentrations of paraquat (**A**); rotenone (**B**); chlorpyrifos (**C**); pendimethalin (**D**); endosulfan (**E**); fenpyroximate (**F**); tebufenpyrad (**G**); trichlorphon (**H**); and carbaryl (**I**) for 12 h, followed by ATP-level detection using a luciferase-based assay and analysis by one-way ANOVA and Dunnett’s test (*n* = 4). Error bars represent SEM. * *p* < 0.05; ** *p* < 0.01; *** *p* < 0.001.

We determined the effect of pesticides on intracellular ATP concentration as a functional parameter of mitochondria. Cells were exposed to low concentrations of pesticides for 12 h and ATP levels were determined using a luciferase-based assay. Dose-dependent ATP depletion was detected in SH-SY5Y cells in response to five pesticide treatments, including paraquat, rotenone, chlorpyrifos, fenpyroximate and tebufenpyrad (Figure 3A–C,F,G). Rotenone was the most potent, with ATP depletion observed at concentrations as low as 10 nmol/L, whereas pendimethalin, endosulfan, trichlorphon and carbaryl were found not to influence cellular ATP (Figure 3D,E,H,I).

### 2.4. Paraquat, Rotenone and Chlorpyrifos Inhibited NADH Dehydrogenase Activity

**Figure 4:**
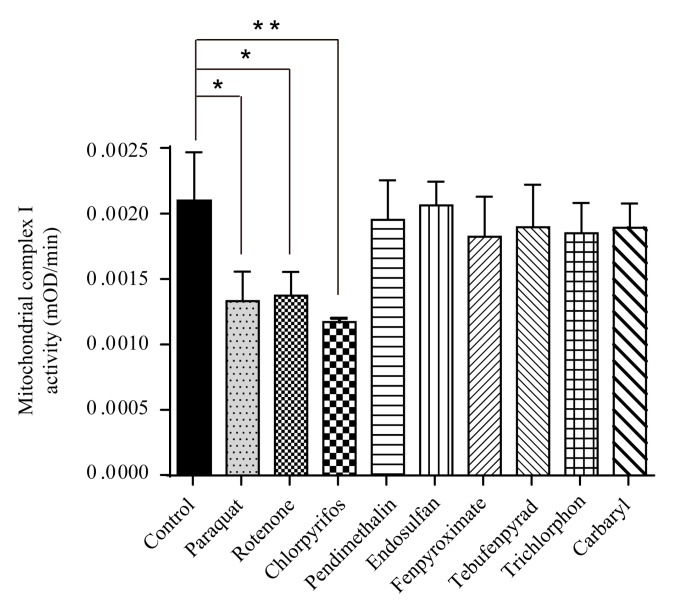
Paraquat, rotenone and chlorpyrifos inhibited NADH dehydrogenase activity. SH-SY5Y cells were exposed to low concentrations of paraquat (0.5 mM), rotenone (100 nM), chlorpyrifos (500 μM), pendimethalin (50 μM), endosulfan (50 μM), fenpyroximate (2.5 μM), tebufenpyrad (50 μM), trichlorphon (200 μM) and carbaryl (100 μM) for 24 h, then NADH dehydrogenase activity was detected and analyzed by one-way ANOVA and Dunnett’s test (*n* = 5). Error bars represent SEM. * *p* < 0.05; ** *p* < 0.01.

NADH dehydrogenase is the first enzyme (complex I) of the mitochondrial electron transport chain, which translocate four protons across the inner membrane per molecule of oxidized NADH, helping to build the electrochemical potential used to produce ATP. As paraquat, rotenone, chlorpyrifos, fenpyroximate and tebufenpyrad caused a significant dose-dependent decrease of intracellular ATP, we examined their effect on NADH dehydrogenase activity. The results showed that NADH dehydrogenase activity was decreased in paraquat- (0.5 mM), rotenone- (100 nM) and chlorpyrifos (500 μM)-treated cells as compared to controls (Figure 4). It is known that chlorpyrifos causes oxidative stress and neurotoxicity in humans and animals [38,39], which may be attributed to the activity disorder of NADH dehydrogenase. Dichlorvos, another organophosphate, caused the same effect through the same mechanism [40]. Paraquat and rotenone are classical complex I inhibitors [24,41]. On the other hand, trichlorphon (200 μM), carbaryl (100 μM), fenpyroximate (2.5 μM), pendimethalin (50 μM), endosulfan (50 μM) and tebufenpyrad (50 μM) had no significant influence on NADH dehydrogenase activity (Figure 4).

### 2.5. All Present-Study Pesticides Except Trichlorphon and Carbaryl Inhibited 26S Proteasome Activity

**Figure 5:**
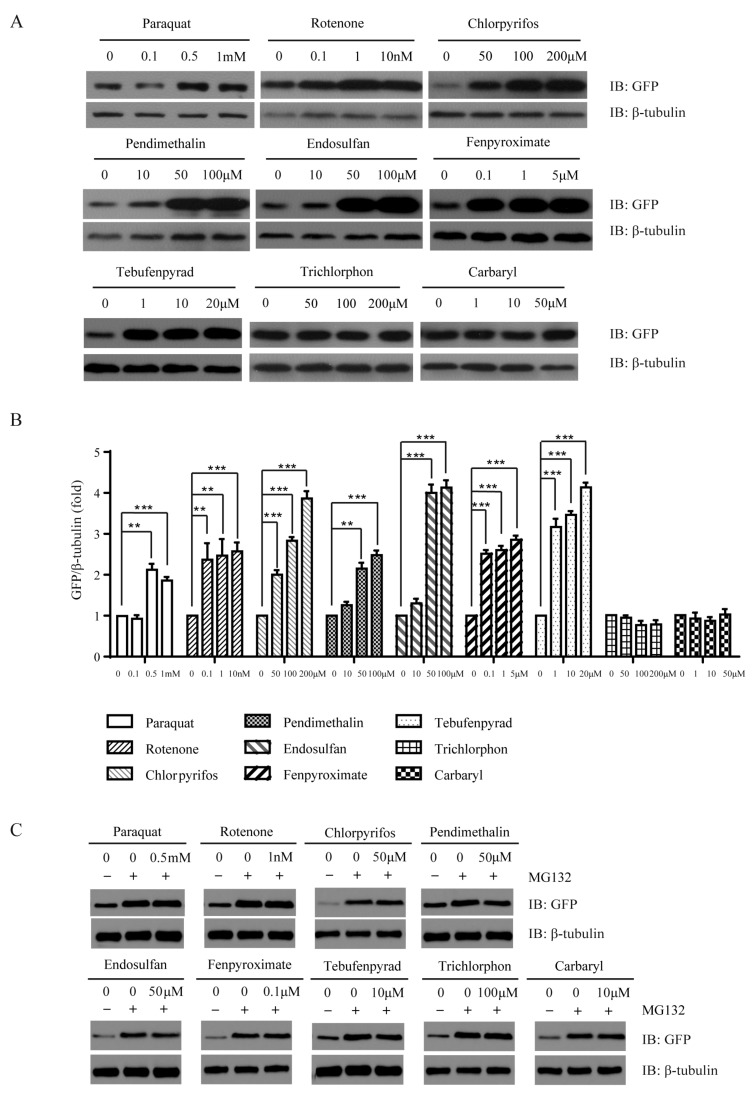
All present-study pesticides except trichlorphon and carbaryl inhibited 26S proteasome activity. SH-SY5Y cells stably expressing GFPU were exposed to low concentrations of pesticides for 24 h, and GFP expression levels were detected by immunoblotting (**A**); relative levels of GFP were measured by ImageJ and analyzed by one-way ANOVA and Dunnett’s test (*n* = 4) (**B**); error bars represent SEM. * *p* < 0.05; ** *p* < 0.01; *** *p* < 0.001. β-tubulin was detected as a loading control. (**C**) SH-SY5Y cells stably expressing GFPU were exposed to pesticides (500 μM paraquat, 1 nM rotenone, 50 μM chlorpyrifos, 50 μM pendimethalin, 50 μM endosulfan, 0.1 μM fenpyroximate, 10 μM tebufenpyrad, 100 μM trichlorphon, 10 μM carbaryl) with 0.1 μM MG132 for 24 h, and GFP expression levels were detected by immunoblotting.

UPS dysfunction is an important pathogenic factor in PD [11,42]. Exposure to pesticide may increase the risk of developing PD by inhibiting the UPS. The effects of pesticides on proteasome activity were examined in SH-SY5Y cells overexpressed with a GFP-conjugated proteasome degradation signal, GFPU [43]. The product of GFPU was continuously degraded and kept at very low levels under normal conditions. Compromised proteasome function reduced the clearance capacity of UPS and increased the steady-state GFPU level. Pesticides were administered to the cultures for 24 h. Expression levels of GFPU were determined by immunoblotting. Our results showed that seven out of nine representative pesticides, namely paraquat, rotenone, chlorpyrifos, pendimethalin, endosulfan, fenpyroximate and tebufenpyrad, had inhibitory effects on proteasome activities at low concentrations (Figure 5A,B). Trichlorphon and carbaryl had no significant influence on proteasome activity (Figure 5A,B). In order to prove that the increased GFPU level with pesticide treatment was the consequence of inhibition of proteasome activities, cells were exposed to pesticides with proteasome inhibitor MG132. Our results showed that there was no further increase of GFPU levels with pesticide treatment (Figure 5C). This indicated that the increased GFPU level with pesticide treatment was due to the inhibition of proteasome activities.

### 2.6. All Present-Study Pesticides Except Trichlorphon and Carbaryl Inhibited 20S Proteasome Activity

**Figure 6:**
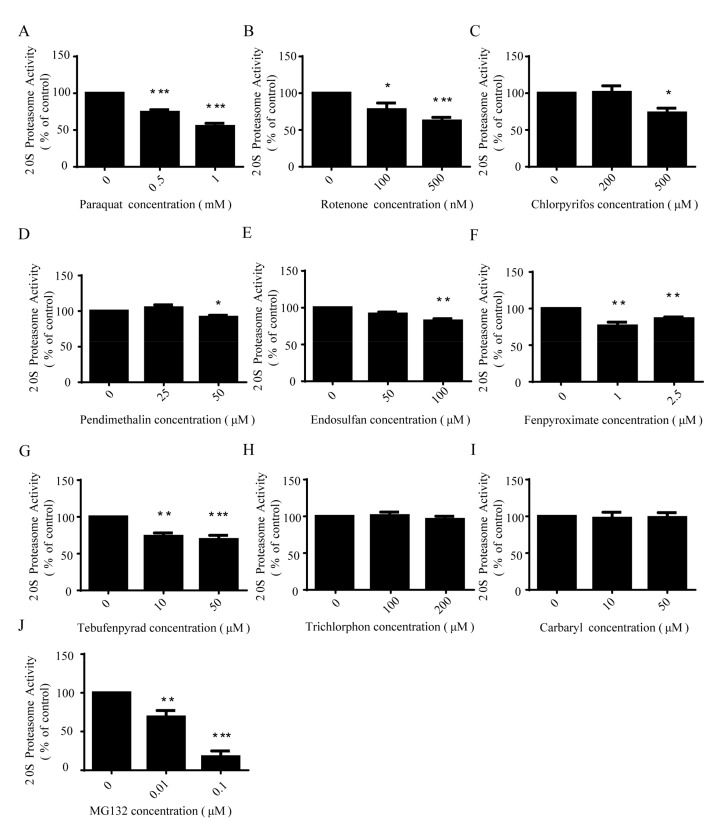
All present-study pesticides except trichlorphon and carbaryl inhibited 20S proteasome activity. SH-SY5Y cells were exposed to low concentrations of paraquat (**A**); rotenone (**B**); chlorpyrifos (**C**); pendimethalin (**D**); endosulfan (**E**); fenpyroximate (**F**); tebufenpyrad (**G**); trichlorphon (**H**); and carbaryl (**I**) for 24 h; MG132 (**J**) was the positive control. The 20S proteasome activity was measured with addition of substrate LLVY-AMC and analyzed by one-way ANOVA and Dunnett’s test (*n* = 5). Error bars represent SEM. * *p* < 0.05; ** *p* < 0.01; *** *p* < 0.001.

To explore the mechanism by which pesticides may inhibit proteasome function, we examined the action of the pesticides on 20S proteasome. SH-SY5Y cells were treated with different concentrations of pesticides for 24 h. Cell lysates were then collected. After collection, the proteasome activity was monitored by the fluorogenic substrate LLVY. Our results showed that seven pesticides, namely paraquat, rotenone, chlorpyrifos, pendimethalin, endosulfan, fenpyroximate and tebufenpyrad, had an inhibitory effect on the 20S proteasome activity (Figure 6). However, there was no inhibition owing to trichlorphon or carbaryl.

### 2.7. Trichlorphon and Carbaryl Did Not Affect Mitochondrial and UPS Functions

According to the above results, five out of the nine representative pesticides, including paraquat, rotenone, chlorpyrifos, fenpyroximate and tebufenpyrad, induced mitochondrial fragmentation and also caused a decrease of intracellular ATP. However, interestingly, among these five pesticides, only paraquat, rotenone and chlorpyrifos inhibited NADH dehydrogenase activity. Moreover, of the nine pesticides, only pendimethalin and endosulfan affected the mitochondrial morphology without affecting the intracellular ATP and NADH dehydrogenase activity. It is noted that the pesticides that affected the morphology and function of mitochondria also inhibited the activity of the 20S and the 26S proteasome.

Most importantly, among these nine presently studied pesticides, trichlorphon and carbaryl were exceptionally found not to cause mitochondrial fragmentation. Furthermore, these two pesticides were determined not to have an effect on the intracellular ATP and NADH dehydrogenase activity in the half-lethal concentration treatments, nor inhibit the proteasome activity. These results suggest that trichlorphon and carbaryl have low toxicity for mitochondrial and UPS functions (Table 1).

## 3. Discussion

The etiology of PD is complex and diverse. Some mutations have been described to lead to familial PD. However, the most common PD cases are sporadic and suspected to be attributed to environmental rather than genetic factors. Epidemiologic studies show that daily habits, dietary factors, drugs, medical history and exposure to toxic environmental agents are associated with risks of PD [31,44,45]. As part of the environmental factors, several pesticides contribute to PD with unknown mechanisms.

Pesticides are a ubiquitous component of our environment and have been widely used in China. In 1999, over 5.6 billion pounds of pesticides were applied worldwide, resulting in detectable levels of pesticides in human bodies [32]. Pesticides can be grouped by their functional class of organisms (e.g., herbicides or insecticides) or their chemical structures. In our study, we have chosen the representative members that cover a wide range of pesticides commonly used in China, including dipyridyl compounds, rotenoids, organophosphates, dinitroanilines, organochlorides, acaricides and carbamates. Epidemiological studies indicate that exposure to pesticides has profound effects on the nervous system, in which high-level exposure to the pesticide is associated with neurodegenerative symptoms as well as deficits in neurobehavioral performance and abnormalities [46,47]. Chronic low-level exposure to pesticides is also associated with increased risk of neurologic disease [32]. The farmers exposed to multiple pesticides have been reported to have a broad range of nonspecific symptoms, such as headache, insomnia and confusion [48,49]. Nevertheless, the neurotoxic mechanisms of chronic low-level exposure have remained unclear. Therefore, our study of the mitochondria and UPS inhibition with a low concentration of pesticide treatment may give enlightenment to the problem.

In the current study, we examined the neurotoxic mechanism of commonly used pesticides in China. The results revealed that pesticides induce morphological changes of mitochondria and dose-dependent apoptotic cell death. Some of the pesticides caused a significant dose-dependent decrease of intracellular ATP and NADH dehydrogenase activity. Moreover, most pesticides inhibited 26S and 20S proteasome activity. Many research findings on sporadic and familial PD cases show that an impairment of mitochondrial function results in age-related neurodegeneration. In addition, several studies indicate that dysfunction of mitochondrial dynamism [50,51], complex I deficiency [52,53], mtDNA mutations [54,55] and blocked mitophagy [56,57] contribute to the pathogenesis of PD. Dysfunctional UPS also contributes to protein aggregation [11,58,59], oxidative stress [60,61] and dopaminergic cell death in Parkinson’s disease pathogenesis. Therefore, our findings suggest that mitochondria and UPS inhibition constitute a potential mechanism for pesticide-induced Parkinsonism.

An exposure to pesticides and mitochondrial dysfunction are increasingly implicated in the pathogenesis of PD. In our study, paraquat, rotenone, chlorpyrifos, fenpyroximate and tebufenpyrad induced mitochondrial fragmentation and ATP decline; however, only paraquat, rotenone and chlorpyrifos inhibited NADH dehydrogenase activity. The research of paraquat toxicity in mouse strains and rats show that it can induce PD-like lesions. The underlying mechanisms are directly or indirectly related to reactive oxygen species [62,63]. Rotenone, fenpyroximate and tebufenpyrad have also been demonstrated to displace 3*H*-dihydrorotenone (DHR) from complex I and inhibit complex I activity [24,34]. Trichlorphon and carbaryl increase the length of mitochondria or the ratio between the bigger (major) and the smaller (minor) side for each mitochondrial fragment, but do not induce mitochondrial functional changes (ATP, NADH dehydrogenase activity and mitochondrial membrane potential). This suggests that trichlorphon- or carbaryl-induced cell toxicity is not mainly through damaging mitochondria. However, the mechanism of trichlorphon- or carbaryl-induced mitochondrial morphology changes needs to be further elucidated.

Interestingly, pesticides that induced mitochondrial fragmentation and ATP decline also inhibited UPS function. It might be due to the 26S proteasome, which is the main component in the UPS, that functions in an ATP-dependent manner [64]. One group of byproducts of aerobic metabolism in mitochondria are reactive oxygen species, and the accumulation of these oxidized proteins is associated with age-related diseases [65,66]. Oxidative stress not only damages the proteins, which are degraded, but also impairs the composition of the UPS itself [67]. Hence, altered UPS function might occur as a secondary response to cell death. On the other hand, the UPS is a part of the surveillance network which controls the mitochondrial protein quality and mitochondrial integrity [64,68]. The UPS itself is also a key cellular event responsible for degeneration, as our results revealed that pendimethalin and endosulfan do not cause a decrease of ATP while inhibiting 20S and 26S proteasome activity. Epidemiological studies suggest that exposure to organophosphorus, carbamate and organochlorine insecticides, as well as dithiocarbamate fungicides, might be associated with PD [7,69,70]. However, except for organophosphorus, the detailed neurotoxic mechanism had remained unclear. The current study elucidates the mechanism of neurotoxicity for these pesticides associated with PD. Although acaricide, fenpyroximate and tebufenpyrad caused neurotoxicity through a similar mechanism, chlorpyrifos and trichlorphon (both organophosphorus) were found to induce cell death in a different manner, indicating that specific pesticides induce different neurotoxicological pathways.

Our results show that, of the nine examined pesticides in this study, exclusively trichlorphon and carbaryl do not inhibit the activity of mitochondria and the ubiquitin-Proteasome system. Trichlorphon and carbaryl are commonly used insecticides in China [71]. Trichlorphon is a member of the organochloride insecticides inhibiting acetylcholinesterase, and has long been used as a pesticide for agricultural plant protection and as a repellent for the treatment of parasitic diseases. Trichlorphon has also been proposed for the treatment of Alzheimer’s disease [72]. As for carbaryl, it is a member of the carbamate family, which are slowly reversible inhibitors that disrupt the cholinergic nervous system and cause death [73]. In general, both trichlorphon and carbaryl may have low toxicity for mitochondrial and UPS functions, and also may cause cell apoptosis through other mechanisms. However, further studies are needed to demonstrate these hypotheses.

In conclusion, our study reveals that some pesticides commonly used in China induce mitochondrial as well as proteasome dysfunction, which might constitute a potential mechanism of the neurodegeneration in Parkinsonism. Hence, our findings provide significant guidance on the subject of pesticide selection for agricultural, industrial and domestic applications. Further studies, including in-vivo animal experiments, are necessary for the determination of the actual risk of pesticides to human health.

## 4. Materials and Methods

### 4.1. Materials

SH-SY5Y and HeLa cells were obtained from American Type Culture Collection (ATCC) and maintained as suggested by the provider. HeLa cells were cultured in Dulbecco’s High Glucose Modified Eagles Medium (GE Healthcare HyClone, Logan, UT, USA, SH30022) containing 2 mM/L l-glutamine (Life Technologies, Waltham, MA, USA, 25030) and 1 mM/L sodium pyruvate (Life Technologies, Waltham, MA, USA, 11360) supplemented with 10% fetal bovine serum (GE Healthcare HyClone, Logan, UT, USA, SH30084). SH-SY5Y cells were cultured in DMEM: F12 (GE Healthcare HyClone, Logan, UT, USA, SH30023) containing 1 mM/L Non-Essential Amino Acids (Thermo Fisher Scientific, Waltham, MA, USA, 11140050), 200 mM l-glutamine (GIBCO, Thermo Fisher Scientific, Waltham, MA, USA, 25030) and 100 mM sodium pyruvate (Thermo Fisher Scientific, Waltham, USA, 11360070) supplemented with 10% fetal bovine serum (GE Healthcare HyClone, Logan, UT, USA, SH30084). The nine tested pesticides, namely paraquat (36541); rotenone (R8875); chlorpyrifos (90047); pendimethalin (36191); endosulfan (PS81); fenpyroximate (31684); tebufenpyrad (46438); trichlorphon (45698); and carbaryl (32055), were all from Sigma-Aldrich (Billerica, MA, USA). Most pesticides were dissolved in dimethyl sulfoxide (DMSO), except for paraquat in H_2_O. DMSO final concentration was not more than 0.1% in the culture medium. A stable cell line expressing GFPU was generated as described [74] and maintained in 200 μg/mL G418.

### 4.2. Cell Apoptosis Assay

Cell apoptosis was quantified using an Annexin V-FITC Apoptosis Detection Kit according to manufacturer’s instruction (Sigma-Aldrich, Billerica, MA, USA, APOAF-20TST). Briefly, after pesticides exposure, cells collected by trypsin digestion were stained by mixing with 3 μL annexin V-FITC and 1 μL propidine iodide (PI), followed by incubation at room temperature in darkness for 15 min. Apoptotic cells were immediately analyzed using a FACScan flow cytometer (Becton Dickinson, SanJose, CA, USA) with excitation at 488 nm and emission at 530 nm (FITC) and 610 nm (PI). Each experiment was repeated three times.

### 4.3. Immunofluorescence and Analysis of Mitochondrial Morphology

Cells were cultured on glass coverslips for 24–48 h, followed by fixation with 4% paraformaldehyde for 10 min and permeabilization with 0.1% Triton X-100 in PBS for another 10 min at room temperature. After blocking with 3% BSA for 30 min, cells were stained with mouse anti-Tom20 monoclonal antibody (Santa Cruz, Dallas, TX, USA, sc-17764) and then incubated with the Goat Anti-Mouse IgG (H + L) Cross-Adsorbed Secondary Antibody, Alexa Fluor 546 (Invitrogen, Waltham, MA, USA, A-11003). Following this procedure, cell nuclei were counterstained with DAPI (Thermo Fisher Scientific, Waltham, MA, USA, D1306), and images were acquired under a Leica confocal microscope (Buffalo Grove, IL, USA, TCS SP5) with appropriate excitation and emission filter pairs. For mitochondrial morphology analysis, aspect ratio (an index of mitochondrial branch length) means the ratio between the bigger (major) and the smaller (minor) side for each mitochondrial fragment. The images were processed to maximize the signal/background ratio using the automatic adjustment of brightness/contrast of the ImageJ software. Finally, the major and the minor of each fragment were determined using the “analyse particles” function of ImageJ. Analyses were performed on at least 50 cells for each condition [75].

### 4.4. Immunoblotting

Cells were lysed with SDS sample buffer (63 mM Tris-HCl, 10% glycerol, 2% SDS). After supernatant collection, protein concentration was determined using a BCA protein assay kit (Thermo Fisher Scientific, Waltham, MA, USA, 23225). Sample proteins were separated on SDS–PAGE gels and transferred onto PVDF membranes. The membranes were blocked with 5% non-fat dry milk in 0.1% Triton X-100/PBS buffer for 1 h and then were incubated with appropriate primary antibodies in blocking solution overnight at 4 °C. Following rinse with 0.1% Triton X-100/PBS buffer, the membranes were re-incubated with appropriate secondary antibodies for 1 h and immunoreactive bands were detected via enhanced chemiluminescence kit according to the manufacturer’s instruction (Thermo Fisher Scientific, Waltham, MA, USA, 32106). The GFP (632381) and β-tubulin (T8328) antibody were purchased from Clontech (Mountain View, CA, USA) and Sigma-Aldrich (Billerica, MA, USA), respectively.

### 4.5. ATP Measurements

Cellular ATP levels in SH-SY5Y cells exposed to pesticides were determined using the CellTiter-GloH Luminescent Cell Viability Assay Kit (Promega, Madison, WI, USA, G7570). In brief, cells were grown in 24-well fluorimeter plates and exposed to various concentrations of the pesticides. Following removal of media, cells were washed with cold PBS and then ATP levels were determined as described in the manufacturer’s protocol. Then, the determined ATP levels were normalized by total protein concentration. Each experiment was repeated three times.

### 4.6. Mitochondrial Complex I Activity Assay

Mitochondrial complex I activity in SH-SY5Y cells exposed to pesticides was determined via Mitochondrial Complex I Activity Assay Kit (Sigma-Aldrich, Billerica, MA, USA, AAMT001). In brief, cells were grown in 10 cm dishes and exposed to pesticides, and then collected and lysed with Mammalian Tissue Lysis/Extraction Reagent (Sigma-Aldrich, Billerica, MA, USA, C3228). Protein concentration was determined using BCA protein assay kit (Thermo Fisher Scientific, Waltham, MA, USA, 23225). Mitochondrial complex I activity, determined as described in the manufacturer’s protocol, was defined as a change in absorbance at 450 nm per minute for each amount of sample. Each experiment was repeated three times.

### 4.7. Determination of Cellular 26S Proteasome Activity

A stable cell line overexpressing GFPU in SH-SY5Y cells was established as a cellular model to test 26S proteasome activity. The reporter gene, which consists of a short tag CL1, is fused to the C-terminus of GFP. CL1, encoding a fragment of amino acids (ACKNWFSSLSHFVIHL), was shown to be a degradation substrate for the ubiquitin-Proteasome system [43]. The expression levels of GFPU after exposure to pesticides were determined by immunoblotting. 

### 4.8. Determination of 20S Proteasome Activity

20S proteasome activity was determined using the Proteasome Activity Assay Kit (BioVision, Milpitas, CA, USA, K245). Briefly, cells were grown in 6-well fluorimeter plates and exposed to various concentrations of pesticides. Following removal of media, cells were washed with cold PBS and 20S proteasome activity was determined according to the manufacturer’s protocol. The determined 20S proteasome activity was then normalized by total protein concentration. Each experiment was repeated three times.

### 4.9. Statistical Analysis

Statistical analyses were performed using GraphPad Prism 5 software. The data are presented as mean ± SEM. Statistical significance between treatment groups against their controls was derived using one-way analysis of variance (ANOVA) followed by Dunnett’s tests. Two-tailed Student’s *t*-test was used to determine the significance of difference between 2 groups.

## 5. Conclusions

Our research reveals that some pesticides commonly used in China induce mitochondrial as well as proteasome dysfunction, which might constitute a potential mechanism of the neurodegeneration in Parkinsonism.

## Figures and Tables

**Table 1:** Summary of the effects of pesticides on mitochondria and the ubiquitin-Proteasome system.

Name	Chemical Class	Mitochondria			Ubiquitin-Proteasome System	
		Morphology	ATP	Complex I	26S	20S
Paraquat	Dipyridyl compounds	Fragmentation	Depletion	Decrease	Inhibition	Inhibition
Rotenone	Rotenoids	Fragmentation	Depletion	Decrease	Inhibition	Inhibition
Chlorpyrifos	Organophosphates	Fragmentation	Depletion	Decrease	Inhibition	Inhibition
Pendimethalin	Dinitroanilines	Fragmentation	No influence	No influence	Inhibition	Inhibition
Endosulfan	Organochlorides	Fragmentation	No influence	No influence	Inhibition	Inhibition
Fenpyroximate	Acaricides	Fragmentation	Depletion	No influence	Inhibition	Inhibition
Tebufenpyrad	Acaricides	Fragmentation	Depletion	No influence	Inhibition	Inhibition
Trichlorphon	Organophosphates	Elongation	No influence	No influence	No influence	No influence
Carbaryl	Carbamates	Elongation	No influence	No influence	No influence	No influence

## Abbreviations

PD	Parkinson’s Disease
PI	Propidine Iodide
UPS	Ubiquitin-proteasome System
WHO	World Health Organization
DHR	[3H]dihydrorotenone
